# Recognition and Resistance as Dual Pathways in Self-Relevant Advertising: The Role of Narcissistic Admiration and Rivalry

**DOI:** 10.3390/bs16040551

**Published:** 2026-04-07

**Authors:** Avi Besser, Virgil Zeigler-Hill, Iris Gertner Moryossef

**Affiliations:** 1 Department of Communication Disorders, Jerusalem Multidisciplinary College, Jerusalem 91010, Israel; 2 Department of Psychology, Oakland University, Rochester, MI 48309, USA; zeiglerh@oakland.edu; 3 School of Management, Jerusalem Multidisciplinary College, Jerusalem 91010, Israel; irisge@jmc.ac.il

**Keywords:** narcissistic admiration, narcissistic rivalry, self-relevant advertising, recognition/validation framing, perceived freedom threat, state reactance, dual-path mediation

## Abstract

This study examined how distinct dimensions of grandiose narcissism shape responses to self-relevant video advertising framed as either recognition/validation or status challenge. Drawing on the Narcissistic Admiration and Rivalry Concept, we tested a dual-path process model in which two proximal mechanisms–perceived recognition and autonomy-related resistance (operationalized as perceived freedom threat and state reactance)–are associated with advertising-related outcomes. Community adults (*N* = 598) were randomly assigned to view one of two video advertisements and subsequently reported perceived recognition, resistance, and consumer responses. Recognition framing increased perceived recognition but did not directly influence consumer outcomes. Process analyses revealed distinct personality-linked patterns that were consistent with the proposed dual-path model. Narcissistic admiration was associated with more favorable attitudes toward the advertisement and brand, as well as stronger purchase intentions, indirectly through higher perceived recognition. In contrast, narcissistic rivalry showed contrasting indirect associations, with positive indirect associations through recognition alongside negative indirect associations through resistance. Moderated mediation by message framing was not supported. Overall, the findings are consistent with the view that self-relevant advertising can simultaneously activate affirmation-related and autonomy-protective processes that may partially offset one another at the aggregate level. Importantly, consumer responses appear to depend on whether the persuasive encounter is construed as authentic recognition or as an autonomy threat–an interpretive dynamic that is especially pronounced among individuals high in narcissistic rivalry.

## 1. Introduction

Self-relevant and identity-based advertising has become a central strategy in contemporary marketing. Rather than emphasizing product attributes, advertisers increasingly craft messages that align with consumers’ self-concepts, values, and desired social identities (Burnkrant & Unnava, 1995; Escalas, 2007). In digital environments, such identity-oriented appeals are often embedded within personalized formats that intensify perceived relevance and psychological involvement (Bang et al., 2019; Yeo et al., 2025). Despite their strategic appeal, the psychological consequences of self-relevant advertising are not uniformly positive. Identity-linked messages may enhance engagement and persuasion in some cases, yet they may also elicit resistance or defensiveness in others. This variability underscores the need to clarify the mechanisms through which self-relevant advertising shapes consumer responses.

The persuasive impact of self-relevant appeals depends less on their formal characteristics and more on how consumers construe their psychological meaning. When experienced as affirming and autonomy-supportive, such messages may foster perceived recognition and strengthen positive evaluations. When construed as evaluative or controlling, however, they may trigger psychological reactance and resistance (Dillard & Shen, 2005; Li & Shi, 2026). This tension suggests that self-relevant advertising may operate through distinct psychological pathways that can enhance or undermine persuasion.

### 1.1. Literature Review

#### 1.1.1. The Persuasive Power of Self-Relevant Advertising

Self-targeted advertising relies on self-referencing processes through which individuals connect message content to their identity, experiences, or personal values. Classic evidence shows that self-referencing increases elaboration and memory for persuasive messages (Burnkrant & Unnava, 1995), particularly when message arguments are strong (Meyers-Levy & Peracchio, 1996). More recent work extends this idea to narrative self-referencing, suggesting that persuasion can operate via transportation, the psychological experience of being absorbed in a story (Escalas, 2007).

In contemporary digital environments, self-targeting is frequently implemented algorithmically through personalized messages that highlight consumers’ preferences or behaviors (Bang et al., 2019). This precision can increase relevance and engagement, but it also intensifies the psychological meaning of the encounter: ads that signal knowledge of the consumer may be experienced as recognition and validation, yet the same cue can also be construed as intrusive and controlling. Consistent with privacy and persuasion knowledge perspectives, reactions to personalization depend strongly on trust and perceived control over how personal information is used (Aguirre et al., 2015; Bleier & Eisenbeiss, 2015; Friestad & Wright, 1994; Tucker, 2014; Zahirović et al., 2024). Thus, self-relevant advertising can elicit both affirmation-related and resistance-related responses, making it essential to examine the mediating roles of perceived recognition and psychological reactance.

Crucially, personalization should be distinguished from recognition. Personalization is a technological delivery mechanism that algorithmically matches content to users based on behavioral or demographic data, whereas recognition is a motivational and psychological experience of being genuinely seen, validated, and understood. Accordingly, perceived recognition is a self-referential appraisal, not a property of targeting technology. The same personalized content may or may not be experienced as recognition, depending on whether it is construed as autonomy-supportive acknowledgment or as intrusive and controlling persuasion; accordingly, personalization is neither necessary nor sufficient for perceived recognition. A data-driven match can therefore be interpreted as caring and affirming or as intrusive and controlling, depending on the perceived intent attributed to the advertiser.

This conceptual distinction is central to understanding self-relevant persuasion and is consistent with research on online behavioral advertising (OBA). Advertisements that signal knowledge of the consumer can enhance relevance and engagement, but they may also elicit privacy concerns, skepticism, and perceptions of manipulation, thereby diminishing persuasive effectiveness (Baek & Morimoto, 2012; Boerman et al., 2017, 2021; Edwards & Lee, 2002; Tucker, 2014). Responses to such messages depend heavily on contextual factors such as trust, transparency, and perceived control over the use of personal information (Aguirre et al., 2015; Bleier & Eisenbeiss, 2015; Tucker, 2014; Xu et al., 2025). Related work on AI-enabled advertising highlights a similar convenience-privacy tension, whereby personalization can increase usability while simultaneously heightening psychological discomfort when privacy concerns are salient (Wang et al., 2023). Taken together, these findings underscore the importance of distinguishing between supportive self-referential experiences (e.g., perceived recognition) and autonomy-related resistance processes. In the present study, these mechanisms are conceptualized as separable pathways through which self-relevant advertising is associated with persuasion outcomes.

#### 1.1.2. Recognition/Validation Versus Status-Challenge Strategies

Building on these insights, two framing strategies in self-relevant advertising can be distinguished by their psychological meaning: recognition/validation and status-challenge. Recognition/validation advertising affirms the consumer’s existing identity by communicating, implicitly or explicitly, that the brand perceives and understands the individual. Importantly, validation refers to a feature of the message (e.g., a framing cue), whereas perceived recognition refers to the recipient’s psychological experience of feeling seen, understood, and affirmatively acknowledged. Thus, although validation may increase the likelihood of perceived recognition, the two constructs are conceptually distinct. When experienced as genuine validation, such messages are likely to support autonomy and relatedness needs and promote more favorable consumer evaluations through heightened perceived recognition (Deci & Ryan, 2000; Weinstein et al., 2020).

In contrast, status-challenge advertising highlights a discrepancy between the consumer’s current state and an aspirational ideal, urging improvement, achievement, or competition. Such appeals are designed to activate self-enhancement and status motives and may be experienced as motivating for some viewers. However, the same evaluative framing can also be construed as pressuring or controlling. When interpreted as evaluative pressure, status-challenge messages can evoke defensiveness and activate a resistance process, expressed as heightened perceived freedom threat and state reactance (J. W. Brehm, 1966; Miller et al., 2007; Rains, 2013).

When consumers perceive that evaluative messaging threatens their freedom to choose or define themselves, they may experience psychological reactance, a motivational state aimed at restoring autonomy (J. W. Brehm, 1966; Dillard & Shen, 2005). In digital environments, perceived control plays a central role in reducing this resistance. For example, allowing users to customize or manage advertising features, such as personalization settings or data sharing controls, can reduce perceived freedom threat and lower state reactance. (Snyder et al., 2025). This evidence supports treating autonomy threat and reactance as a coherent resistance pathway in self-relevant persuasion contexts.

Importantly, this distinction rests on the psychological meaning and perceived intent of the message, not on self-relevance or personalization per se. Although both strategies employ self-relevant cues, they differ in whether those cues are framed as supportive validation of the self or as evaluative pressure. This distinction is consistent with research on motivational communication showing that autonomy-supportive framing tends to increase receptivity, whereas controlling or pressuring framing is associated with greater autonomy threat and resistance (Deci & Ryan, 2000; Weinstein et al., 2020). Evidence from reactance research similarly indicates that perceived freedom threat is associated with heightened psychological reactance and reduced persuasion (Dillard & Shen, 2005; Miller et al., 2007; Quick & Stephenson, 2008; Li & Shi, 2026). At the same time, affirmation and resistance are not necessarily mutually exclusive in self-relevant advertising. Depending on how message intent is construed, recognition and resistance may co-occur and function as separable pathways that are jointly associated with downstream consumer evaluations.

#### 1.1.3. Psychological Reactance and Autonomy Threat

Psychological reactance theory provides a central framework for understanding why self-relevant messages may evoke resistance when they are experienced as threatening freedom of choice or self-definition (S. S. Brehm & Brehm, 1981; Miron & Brehm, 2006; Rains, 2013). Messages that convey pressure or control tend to elicit negative affect, oppositional motivation, and less favorable evaluations of the source, thereby undermining persuasive goals (Miller et al., 2007). Meta-analytic evidence further indicates that perceived freedom threat is reliably associated with increased reactance and, in turn, reduced persuasion (Li & Shi, 2026).

Self-Determination Theory (SDT) offers a complementary perspective by explaining why autonomy-supportive and affirming messages are more likely to facilitate persuasion (Deci & Ryan, 2000). When communication is experienced as supportive rather than controlling, it is more likely to satisfy basic psychological needs and promote receptivity and internalization. In the context of self-relevant advertising, this suggests that recognition/validation framing may be associated with greater persuasion through heightened perceived recognition, whereas evaluative or pressuring framing may be associated with reduced persuasion through autonomy-related resistance (Weinstein et al., 2020).

Accordingly, the present study examines recognition and resistance in tandem rather than assuming that self-relevant advertising is uniformly affirming or uniformly controlling. In this framework, resistance is operationalized as a composite index capturing two closely related post-exposure reactions: perceived freedom threat and state reactance. This operationalization follows the theoretical sequence in which perceived freedom threat functions as a proximal trigger of reactance, while also providing a parsimonious observed-variable representation of autonomy-related resistance in response to self-relevant advertising.

#### 1.1.4. Narcissistic Self-Regulation in Self-Relevant Persuasion

Consumers do not uniformly interpret self-relevant messages. Research on grandiose narcissism highlights systematic individual differences in how people regulate self-esteem and respond to social evaluation. Contemporary models conceptualize narcissism as a dynamic self-regulatory system that balances ego-affirming and ego-protective motives, producing shifts between self-promotion and self-defense depending on contextual cues (Weiss et al., 2020). These regulatory dynamics are especially relevant in persuasion contexts, where the same self-relevant message may be construed either as validating recognition or as evaluative pressure, thereby shaping perceived recognition and resistance processes in divergent ways (Campbell & Foster, 2007). Within the Narcissistic Admiration and Rivalry Concept (NARC), grandiose narcissism is differentiated into two related but distinct dimensions–narcissistic admiration and narcissistic rivalry–each reflecting a characteristic self-regulatory orientation (Back et al., 2013).

Narcissistic admiration reflects an assertive, self-enhancing strategy characterized by confidence, social charm, and a desire for uniqueness and recognition. Individuals high in narcissistic admiration tend to pursue social acclaim through self-promotion and are often described as socially potent and extraverted (Back et al., 2013). In contrast, narcissistic rivalry reflects a more antagonistic and defensive strategy marked by entitlement, devaluation of others, and hostility toward perceived competitors. It is associated with low agreeableness and heightened sensitivity to ego-threat and status competition (Back et al., 2013; Rogoza et al., 2019; Stucke & Sporer, 2002).

These distinct regulatory orientations have important implications for how self-relevant messages are processed. Narcissistic admiration, with its strong motivation for affirmation and distinction, should be particularly aligned with recognition-oriented processing of self-relevant cues. Narcissistic rivalry, by contrast, reflects heightened vigilance to status and autonomy threats and should therefore be more likely to engage resistance processes when messages are construed as pressuring or controlling. Consistent with this logic, individuals high in narcissistic rivalry demonstrate greater proneness to reactance, especially when autonomy threats are interpreted as challenges to dominance or standing (Liu & Zheng, 2025).

At the same time, narcissistic rivalry should not be conceptualized as uniformly resistant. Empirical work from social-interaction paradigms indicates that narcissistic admiration and narcissistic rivalry produce distinct patterns of perception and behavior across contexts (Geukes et al., 2017). Recent evidence further suggests that narcissistic rivalry is characterized by heightened vigilance to status-relevant cues broadly construed, including both signals of recognition and cues implying potential loss of standing or evaluative threat (Besser et al., 2026). This pattern implies a more ambivalent processing style in self-relevant contexts: individuals high in narcissistic rivalry may register recognition cues as self-diagnostic and status-affirming while simultaneously remaining alert to possible evaluative pressure. Recognition and defensiveness may therefore co-occur rather than operate in isolation.

These dual motivational tendencies imply that the same self-targeted advertisement can activate different psychological processes across individuals, depending on which narcissistic dimension is more prominent. Recognition/validation appeals are especially likely to increase perceived recognition among individuals high in narcissistic admiration, given their strong motivation for social acclaim. For individuals high in narcissistic rivalry, recognition cues may still register as self-relevant. However, they are more likely to be processed through a status-vigilant and threat-sensitive lens. As a result, recognition can co-occur with heightened sensitivity to evaluative intent and autonomy pressure. Accordingly, rather than assuming that recognition is irrelevant for rivalry, the present study treats perceived recognition and resistance as separable processes that can vary in strength within the same persuasive encounter (Back et al., 2013; Dillard & Shen, 2005; Hart et al., 2021).

A similar logic applies to status-challenge appeals. Framing self-relevance in competitive terms may resonate with dominance and status motives characteristic of narcissistic rivalry, potentially increasing engagement when the challenge is interpreted as an opportunity for advancement. However, the same evaluative framing may also be construed as controlling, thereby activating autonomy-related resistance. For narcissistic admiration, status-challenge framing may be less aligned with a preference for affirmation, yet it may still enhance perceived recognition if interpreted as acknowledging one’s potential. Accordingly, rather than presuming that status-challenge messages are uniformly energizing for narcissistic rivalry and uniformly aversive for narcissistic admiration, the present study tests how narcissistic admiration and narcissistic rivalry map onto recognition and resistance processes in response to both advertising strategies.

These processes are not necessarily mutually exclusive. Recognition and resistance can co-occur within the same persuasive encounter, particularly when status-relevant cues are simultaneously construed as validating and as pressuring. Accordingly, the present study emphasizes the two mediating mechanisms as separable pathways. It further evaluates whether message framing shifts their relative strength, treating any moderation of trait-to-process links as exploratory.

#### 1.1.5. Integrating Theories: Toward a Dual-Path Process Framework

By synthesizing insights from self-referencing, psychological reactance theory, Self-Determination Theory (SDT), and narcissism research, we develop a process-oriented framework to explain how personality and message framing jointly shape responses to self-relevant advertising. Specifically, we examine how trait-level narcissistic motives and message-level strategy are linked to two proximal psychological mechanisms, perceived recognition and perceived freedom threat/state reactance, operationalized as a resistance index. These mechanisms, in turn, predict downstream persuasion outcomes.

At the *trait level*, narcissistic admiration and narcissistic rivalry reflect distinct self-regulatory orientations related to self-validation and self-protection. At the message level, recognition/validation and status-challenge strategies differ in how self-relevance is framed, as supportive acknowledgment versus evaluative challenge. At the *process level*, perceived recognition and perceived freedom threat/state reactance, operationalized as a resistance index, represent two proximal mechanisms through which traits and message framing shape downstream consumer evaluations. These outcomes include attitudes toward the advertisement and brand, as well as purchase intentions.

This framework proposes that narcissistic admiration will be linked to more favorable consumer responses via heightened perceived recognition. Narcissistic rivalry, in contrast, is expected to be associated primarily with resistance processes, expressed as heightened perceived freedom threat and state reactance, which should undermine persuasion. We further examine whether advertising strategy (recognition/validation vs. status-challenge) changes the strength of these links, treating moderation as an empirical question. Accordingly, the central focus is on the two mediating processes and their downstream implications. The framework does not assume that message strategy will reliably amplify trait-based effects.

The result is a dual-path process model in which trait-level narcissistic motives and message framing are linked to two separable mechanisms, perceived recognition and the resistance index, that jointly shape downstream consumer evaluations. The model does not posit an interaction between narcissistic admiration and narcissistic rivalry. Instead, each dimension is examined as a focal predictor in parallel models, while statistically accounting for the other to isolate its unique association with recognition and resistance processes. Advertising strategy is treated as a message-level factor that may shift these processes. Whether it moderates trait-to-mediator links is examined empirically rather than assumed.

#### 1.1.6. Summary and Research Rationale

Taken together, narcissistic admiration and narcissistic rivalry reflect distinct self-regulatory orientations that may shape how self-relevant advertising is interpreted. Narcissistic admiration is more closely aligned with recognition-related processing, whereas narcissistic rivalry is more likely to engage autonomy-related resistance when messages are construed as pressuring or controlling. At the same time, narcissistic rivalry remains highly self-referential and status-focused, making it plausible that recognition and resistance may co-occur within the same persuasive encounter.

Despite substantial advances in research on self-referencing, psychological reactance, and narcissistic self-regulation, these studies have rarely been integrated within a single process model of self-relevant advertising. Prior work has typically examined these components in isolation, leaving limited evidence regarding how they operate together when consumers encounter identity-relevant persuasive messages.

The present study addresses this gap by testing a dual-path process model in which perceived recognition and autonomy-related resistance are jointly associated with downstream consumer evaluations. More specifically, the study integrates narcissistic admiration and rivalry into a framework that examines whether the same self-relevant encounter can simultaneously support affirmation-related and autonomy-protective dynamics. Advertising strategy is incorporated as a message-level factor, and its potential moderation of trait-to-process links is treated as an empirical question rather than assumed in advance.

#### 1.1.7. Conceptual Model

Building on the theoretical synthesis above, the present research proposes a dual-path process model linking distinct dimensions of grandiose narcissism to persuasion outcomes through two psychological mechanisms: perceived recognition and resistance (perceived freedom threat and state reactance). Although both admiration and rivalry represent facets of grandiose narcissism, they reflect different self-regulatory orientations that are theorized to map onto these processes in systematic ways.

Narcissistic admiration is characterized by approach-oriented striving for uniqueness and validation, orienting individuals toward recognition-related processing of affirming self-relevant cues. Narcissistic rivalry is characterized by defensiveness and heightened vigilance to status and autonomy threats, which should increase the likelihood of resistance when messages are construed as pressuring or controlling. At the same time, because rivalry remains strongly self-referential and status-focused, status-relevant cues may also register as recognition cues, allowing recognition and resistance processes to co-occur.

Accordingly, the model posits two separable mediation processes through which grandiose narcissism may shape responses to self-relevant advertising. First, a recognition pathway in which higher narcissistic admiration predicts higher perceived recognition, which in turn is associated with more favorable attitudes toward the advertisement and brand and stronger purchase intentions. Second, a resistance pathway in which higher narcissistic rivalry is expected to predict higher perceived freedom threat and state reactance (resistance index), which in turn should be associated with less favorable consumer responses. Given that rivalry is also strongly status-focused and self-referential, it may simultaneously relate to perceived recognition, yielding potentially competing indirect effects via recognition and resistance. Advertising strategy (recognition/validation vs. status-challenge) is examined as a message-level factor that may shift these processes, and its moderating role is treated as an empirical question.

Within this framework, advertising strategy functions as a message-level factor that can shift the psychological meaning of self-relevant cues and thereby influence perceived recognition and resistance. By integrating individual differences in narcissism with message framing, the model clarifies how identity-based persuasion can operate through affirmation-related processes (perceived recognition) as well as autonomy-related resistance (perceived freedom threat and state reactance), and how these processes translate into consumer evaluations.

In summary, the proposed model integrates trait-level differences in narcissistic admiration and rivalry, a message-level manipulation of advertising strategy, and two process-level mechanisms–perceived recognition and resistance–into a unified framework. By doing so, it specifies not only whether personality is associated with persuasion outcomes, but how these associations are linked to recognition and resistance processes. The model therefore conceptualizes self-relevant advertising as capable of eliciting separable affirmation-related and autonomy-protective responses within the same persuasive encounter.

**Figure 1:**
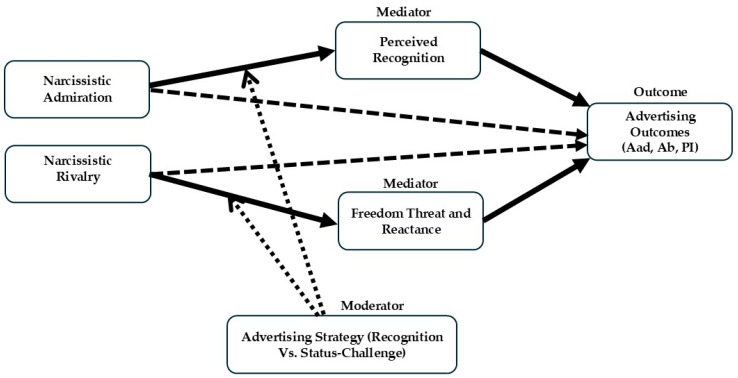
Proposed dual-path process model linking narcissistic admiration and narcissistic rivalry to advertising outcomes via perceived recognition and a resistance index (perceived freedom threat and state reactance). Advertising strategy (recognition/validation vs. status-challenge) is included as a message-level factor, and its potential moderation of the trait-to-mediator paths is examined as an exploratory boundary condition. Solid arrows represent the hypothesized mediation pathways. Dashed arrows represent direct effects on advertising outcomes. Dotted arrows represent the hypothesized moderating effects of advertising strategy (recognition vs. status-challenge) on the predictor-to-mediator paths.

Figure 1 summarizes the proposed dual-path process model and guides the hypotheses that follow.

Against this theoretical background, the present study tests a dual-path process model specifying how self-relevant advertising elicits perceived recognition and resistance, and how these processes translate into persuasion outcomes across recognition/validation versus status-challenge message framing.

### 1.2. Hypotheses

**H1.**  *Recognition/validation advertising will elicit higher perceived recognition than status-challenge advertising (Deci & Ryan, 2000; Weinstein et al., 2020).*

**H2.**  *Narcissistic admiration will be positively associated with perceived recognition (Back et al., 2013).*

**H3.**  *Perceived recognition will mediate the association between narcissistic admiration and persuasive outcomes, such that higher narcissistic admiration will predict more favorable attitudes toward the advertisement and brand and greater purchase intentions via heightened perceived recognition (Weinstein et al., 2020).*

**H4.**  *Narcissistic rivalry will be positively associated with the resistance index (perceived freedom threat and state reactance) (Back et al., 2013; Liu & Zheng, 2025).*

**H5.**  *The resistance index (perceived freedom threat and state reactance) will mediate the association between narcissistic rivalry and persuasive outcomes, such that higher narcissistic rivalry will predict less favorable attitudes toward the advertisement and brand and lower purchase intentions via heightened resistance (Dillard & Shen, 2005; Rains, 2013).*

**H6.**  *The overall pattern of effects will reflect a dual-path process in which perceived recognition is positively associated with persuasion outcomes, whereas the resistance index is negatively associated with persuasion outcomes. Accordingly, narcissistic admiration is expected to show a positive indirect association with consumer outcomes via perceived recognition, whereas narcissistic rivalry is expected to show a competing indirect pattern, with a positive indirect association via perceived recognition alongside a negative indirect association via the resistance index.*

H1 addresses the expected mean-level difference in perceived recognition, whereas H2–H6 examine the primary process-level associations implied by the dual-path framework. In contrast, the moderation analyses are not treated as core confirmatory hypotheses but rather as exploratory boundary-condition tests, given mixed theoretical possibilities regarding whether message framing alters the trait-to-mediator links. The model is tested using conditional process analysis (Hayes, 2022) to estimate indirect effects through each pathway.

## 2. Materials and Methods

### 2.1. Participants

A total of 718 invitations were distributed across the four recruitment cells, yielding 652 completed responses prior to data screening and exclusion procedures. Participants were recruited through iPanel, a well-established local online panel in which individuals voluntarily register to complete surveys in exchange for monetary compensation, and through community-based recruitment channels. Response rates across the four recruitment cells ranged from 86.7% to 95.9%, with an overall response rate of 90.8%. Eligibility criteria included sufficient Hebrew proficiency to complete the online questionnaires and the ability to view video stimuli with audio enabled. Participation was voluntary, and compensation was provided in accordance with panel norms (8 ILS; approximately 2 USD). This recruitment strategy was intended to obtain a broad adult community sample rather than a population-representative one. Accordingly, the sample should not be interpreted as nationally representative, and the findings should be generalized only with caution beyond similar adult community samples. All data were collected via a secure online platform. After providing informed consent, participants completed a brief set of questionnaires, watched a video stimulus, and then completed additional questionnaires. Participants were informed that they could discontinue at any point and that no personally identifiable data would be recorded.

Predetermined exclusion criteria were applied. Participants were removed if they were identified as univariate outliers (i.e., scores more than three standard deviations above or below the mean on one or more study variables; *n* = 15), exhibited highly erratic or internally inconsistent response patterns as operationalized by unusually large variability in their responses across items within a scale (i.e., standardized inter-item standard deviation > 3 for one or more variables; *n* = 28), or showed invariant response behavior (i.e., “straightlining”) as detected via long-string analysis (*n* = 10). Long-string analysis was applied separately to each multi-item measure, and exclusions were based on the observed pattern across measures such that participants were excluded if three or more measures contained identical responses spanning at least 90% of the items within that measure. These screening criteria were defined prior to the final analyses and are also documented in the OSF repository.

The final analytic sample included 598 adults (295 men, 303 women). Participants were randomly assigned to view either the recognition/validation advertising strategy condition (*n* = 304; 149 men, 155 women) or the status-challenge advertising strategy condition (*n* = 294; 146 men, 148 women). Across conditions, the videos were matched on overall autonomy-supportive tone and length; the manipulation targeted the strategic meaning conveyed about the viewer’s status and self-worth.

Participants ranged in age from 20 to 65 years (M = 42.83, SD = 12.58). On average, they reported having 1.90 children. With respect to digital video advertising exposure participants reported moderate frequency of exposure to digital video ads (M = 4.94, SD = 1.80), moderate level of intensity of social media use (M = 4.82, SD = 1.91), and moderate level of frequency of online shopping (M = 4.81, SD = 1.70).

Educational attainment was generally high: 13.2% reported less than a high school education, 23.9% completed high school, 41.8% held a bachelor’s degree, 18.9% held a master’s degree, and 2.2% held a doctoral degree or equivalent. Regarding employment status, most participants reported full-time employment (70.4%), followed by part-time employment (18.6%). Smaller proportions reported being unemployed (3.8%), enrolled in school (2.0%), retired (2.0%), or being a homemaker (1.5%).

Most participants were married (62.0%), with others reporting being divorced (7.0%), widowed (0.7%), single (15.9%), cohabiting (7.2%), separated (0.5%), or dating (6.7%). Household income was broadly distributed, with 14.4% reporting very high income, 21.4% somewhat high, 26.9% moderate, 20.9% somewhat low, and 16.4% very low income. Finally, the majority of participants identified as secular (50.0%), followed by traditional (22.6%), religious (13.5%), and ultra-orthodox (13.9%).

A priori power analysis (G*Power 3.1) indicated that a minimum of 395 participants would be sufficient to detect a small moderated-mediation effect (f^2^ = 0.02, α = 0.05, 1 − β = 0.80). The final sample (*N* = 598) therefore provided excellent statistical power (>0.99) for all planned analyses.

### 2.2. Materials

#### 2.2.1. Video Stimuli

Four brief video advertisements served as the experimental stimuli. To accommodate Hebrew grammatical gender, two narrator-gender versions (male vs. female) were produced for each advertising strategy, resulting in four finalized videos. Each participant viewed one advertisement only, selected from this set of four standardized versions (2 message strategies × 2 narrator-gender versions). Participants were randomized to advertising strategy (recognition/validation vs. status-challenge). Narrator gender was matched to participant gender to preserve linguistic naturalness and identification, such that male participants viewed the male-narrator version and female participants viewed the female-narrator version. Narrator gender was not treated as an experimental factor and was implemented as a design constraint to avoid introducing additional variance attributable to gender-marked Hebrew phrasing or narrator–listener mismatch; critically, within each participant gender, the only between-condition difference was message framing (recognition/validation vs. status-challenge). This constraint helped ensure that post-exposure mediator responses reflected the intended strategic framing rather than incidental linguistic naturalness effects. The focal manipulation remained solely the advertising strategy.

All four clips depicted an identical scenario promoting the same fictitious product and brand, held constant across conditions. The videos were matched on duration (approximately 30 s), setting, camera angle, dialog structure, pacing, and visual design. The only systematic difference between the two strategy conditions was the message framing.

In the recognition/validation condition, the script communicated affirmation and validation, conveying that the brand “saw” and understood the viewer. The language was autonomy-supportive and non-evaluative. In the *status-challenge condition*, the script communicated evaluative comparison and implied discrepancy, emphasizing achievement and status standards that could be construed as challenging. The script avoided direct overlap with mediator measures and did not use the same wording later used in the measures of perceived recognition, freedom threat, or reactance.

**Figure 2:**
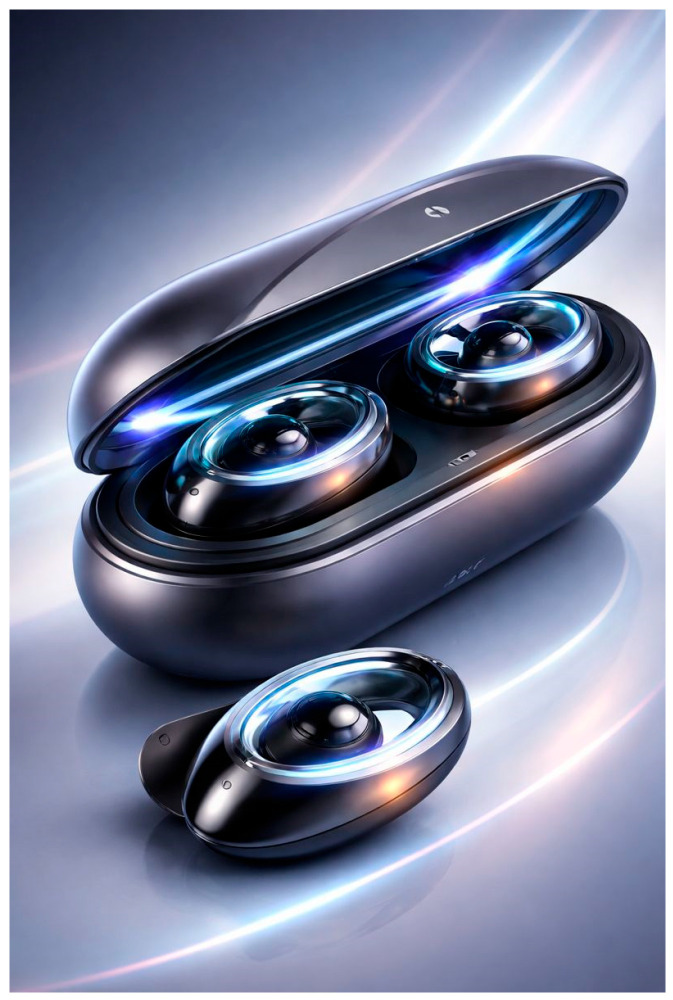
Experimental stimuli depicting the fictitious NOVA AURA wireless earbuds featured in the recognition/validation and status-challenge video advertisements.

A concept illustration of the fictitious product design is provided in Figure 2.

##### Video Advertisements (Stimuli) and Rationale

Participants viewed a short, professionally styled video advertisement created specifically for this experiment. The ad promoted a fictitious premium wireless earbud product (NOVA AURA) designed to be visually plausible yet not resemble any existing commercial model, thereby reducing confounds related to prior brand attitudes, ownership, price knowledge, and idiosyncratic product associations. Using a fictitious brand and product design increased experimental control while preserving ecological validity as a realistic digital advertisement. To further isolate the effects of message framing, a single product category and a standardized short-form video format were used. This design choice minimized additional sources of variance associated with differences in product involvement, prior category beliefs, brand familiarity, and format-specific processing demands, allowing for a more precise test of the focal manipulation.

The experimental manipulation consisted of two advertising strategies: a recognition/validation strategy versus a status-challenge strategy. Importantly, the two ads were matched on all nonfocal dimensions, including product category, storyboard, shot count and shot types, on-screen graphics, pacing, background music, audio levels, and the factual feature information conveyed. Across conditions, the voice-over delivery (pace, intensity, and overall vocal affect) was held constant; only the message framing and wording differed to convey either recognition/validation affirmation or status-challenge/evaluative pressure. This strict standardization strengthens internal validity by ensuring that any between-condition differences in perceived recognition, resistance, and advertising outcomes are attributable to the intended strategy manipulation rather than to differences in content, production quality, or visual esthetics.

To further minimize unintended variance, the ads used a product-only format without a visible spokesperson or identifiable face. This choice reduced the likelihood that actor-specific cues (e.g., attractiveness, age, warmth, interpersonal style) would influence participants’ responses, which is particularly important given that the mediators capture subjective experience (recognition) and resistance processes (perceived freedom threat and reactance).

The ad followed a fixed six-shot structure typical of contemporary digital video advertising: an opening packshot; brief usage-oriented B-roll without faces; close-ups of the product and case; a visual feature demonstration; a benefits montage using icons and minimal text; and a closing packshot with brand mark and tagline.

##### Video Length and Technical Specifications

Each advertisement lasted approximately 30 s and was presented in 16:9 format at 1080p resolution. Background music and overall audio levels were held constant across conditions. Voice-over narration was recorded in both male and female versions. Scripts were matched in length and structure within each condition; apart from grammatical gender marking, wording was held constant.

#### 2.2.2. Final Stimulus Set and Standardization

During stimulus development, multiple candidate renderings of the same scripted advertisement were generated using the AI tools listed below. Participants were not exposed to tool-specific versions. Instead, a single final clip per experimental condition for each narrator gender was selected and exported for experimental use, resulting in four finalized videos (2 conditions × 2 narrator genders). Each participant viewed one full-screen clip only, and therefore the experimental manipulation was solely the advertising strategy.

The finalized videos were used consistently across participants within each condition. All scenario parameters were held constant across conditions except for the intended framing differences.

#### 2.2.3. AI Video-Generation Tools and Production Pipeline

To create high-fidelity, realistic stimuli while holding content constant, a multi-platform AI pipeline was used. These tools were used instrumentally to achieve stimulus realism and strict standardization; the study does not evaluate, compare, or draw inferences about the platforms themselves. Candidate videos were generated using Veo 3.1 (Google DeepMind, London, UK), Kling AI (Kuaishou Technology, Beijing, China), Sora 2 (OpenAI, San Francisco, CA, USA), Pictory, (Bothell, WA, USA), InVideo AI, (InVideo, San Francisco, CA, USA), Runway Gen-3 (Runway AI, New York City, NY, USA), HeyGen (HeyGen Technology Inc., Los Angeles, CA, USA), and Hailuo AI (MiniMax, Shanghai, China). Participants were not exposed to tool-specific versions; rather, a single finalized clip per condition and narrator gender was selected for experimental use.

For each tool, the same prompts, storyboard, reference frames, dialog scripts, target video length, aspect ratio (16:9), and resolution (1080p) were applied. This multi-platform generation pipeline was used instrumentally to achieve high stimulus realism and standardization, not to compare platforms or algorithmic outputs. The narrator’s appearance, camera framing, and background environment were constrained to be as similar as technically possible. Following generation, clips underwent post-processing only when needed to ensure equivalent comprehensibility and timing across conditions. Any adjustment was applied identically across the two strategy conditions and documented in the OSF repository (see Section 2.8), which includes the storyboard, production prompts, and stimulus development notes.

##### AI Content Disclosure

All procedures involving AI-generated materials followed contemporary American Psychological Association guidance on responsible AI use, with emphasis on transparency, data protection, and human oversight in AI-assisted stimulus development and quality assurance (American Psychological Association, 2025; APA Services, 2024).

The video stimuli were synthetically generated using AI tools (e.g., Veo, Runway, Sora) based on original researcher-written scripts and storyboards, developed solely for this study to ensure experimental control and standardization.

No real human likenesses, copyrighted materials, or identifiable content were used. All AI-generated outputs were reviewed and approved by the research team to confirm compliance with ethical and methodological standards.

##### Manipulation Validation (Expert Ratings)

To minimize demand characteristics and avoid conceptual overlap with the post-exposure mediators, no participant-level manipulation-check items were administered. Instead, the manipulation was validated using independent expert ratings. This approach was adopted specifically to avoid contaminating the focal mediators (perceived recognition and resistance) with face-valid manipulation checks that would use overlapping language or prime the intended mechanisms. Judges were blind to the study’s hypotheses and were not informed of the intended condition labels of the advertisements. An expert panel of six judges (three men and three women) with expertise in psychology, marketing and consumer behavior, and communication evaluated the finalized advertisements. Each judge viewed one recognition/validation advertisement and one status-challenge advertisement; to preserve linguistic naturalness in Hebrew, judges viewed the narrator-gender version matched to their own gender, to avoid introducing an additional source of systematic variance related to narrator–listener gender mismatch.

Order of exposure was counterbalanced across judges (half viewed the recognition/validation clip first and half viewed the status-challenge clip first) to minimize potential order or carryover effects. Judges rated each clip on 0–100 scales capturing (a) the extent to which the ad conveyed recognition/validation, (b) the extent to which the ad conveyed status-challenge/evaluative pressure, and (c) the overall autonomy-supportive versus controlling tone (higher scores indicate a more autonomy-supportive tone). For each dimension, the 0 and 100 anchors were defined in advance (0 = not at all; 100 = extremely), and judges were instructed to base ratings on the ad’s strategic content rather than general production quality. Judges completed ratings independently and without discussion. In addition, the autonomy-supportive tone ratings were included specifically to verify that the manipulation differentiated strategic framing while not introducing an overall difference in perceived tone. Inter-rater reliability was assessed using intraclass correlation coefficients. Because the same expert panel rated both advertisements, ICC(3,1) (two-way mixed-effects, consistency) was selected as the appropriate reliability index.

Agreement was high for recognition/validation ratings (ICC = 0.94) and for status-challenge/evaluative pressure ratings (ICC = 0.96). Because the two advertisements were intentionally matched on overall autonomy-supportive tone, between-ad variance on this dimension was minimal, rendering ICC estimates for tone uninformative; equivalence on tone was therefore evaluated using paired-samples t-tests.

As expected, the recognition/validation advertisement was rated substantially higher on recognition/validation (M = 91.33, SD = 2.25) than the status-challenge advertisement (M = 58.33, SD = 7.53), t(5) = 9.77, *p* < 0.001, CI_95%_ [24.32, 41.68], Cohen’s dz = 3.99. Conversely, the status-challenge advertisement was rated substantially higher on status-challenge/evaluative pressure (M = 63.33, SD = 8.16) than the recognition/validation advertisement (M = 25.00, SD = 10.49), t(5) = 12.47, *p* < 0.001, CI_95%_ [30.43, 46.23], Cohen’s dz = 5.09. Importantly, the advertisements did not differ in overall tone (recognition/validation: M = 88.00, SD = 5.10; status-challenge: M = 87.83, SD = 2.48), t(5) = 0.09, *p* = 0.935, CI_95%_ [−4.82, 5.15], Cohen’s dz = 0.04. This expert-based validation approach verified that the manipulation produced strong differences in strategic content (recognition/validation vs. status-challenge) while holding the general tone constant, thereby supporting the interpretation that downstream participant differences reflected the intended strategy framing rather than unintended differences in overall communicative tone.

Because the advertisements were intentionally matched on overall autonomy-supportive tone, we did not expect the status-challenge condition to uniformly elevate post-exposure resistance at the mean level. Instead, we expected resistance to vary primarily as a function of individual differences in threat appraisal, particularly narcissistic rivalry, which the conditional process models were designed to capture. At the same time, expert-based validation does not fully substitute for participant-level evidence regarding how the advertisements were construed by respondents. Because no participant-level manipulation-check items were administered, the present study cannot directly verify subjective framing interpretations independently of the focal mediators. Although this decision reduced the risk of conceptual contamination of the mediators, it also limits the strength of inferences that can be drawn about the effectiveness of the manipulation.

### 2.3. Procedure

After providing informed consent, participants completed demographic background questions and the narcissism questionnaire. Participants were then instructed to watch the video advertisement carefully. Each participant was randomly assigned to one of the two advertising strategy conditions and viewed a gender-matched version of the assigned advertisement. The video clip was presented full-screen, and participants were not able to skip ahead. This standardized presentation format was used to maximize experimental control, although it necessarily differs from more naturalistic digital media environments in which consumers encounter multiple competing stimuli and distractions.

Immediately after viewing the clip, participants completed the post-manipulation measures in the following order: perceived recognition, perceived freedom threat, state reactance, and advertising outcomes (Aad, Ab, PI). Finally, participants completed quality-control items and debriefing materials.

### 2.4. Measures

#### 2.4.1. Demographic Questionnaire and Background Variables

Participants reported age, gender, education, employment status, relationship status, perceived socioeconomic status, and religiosity. Given the advertising context, participants also reported background variables relevant to digital video advertising exposure, including frequency of exposure to digital video ads [“To what extent are you exposed to digital video advertisements (e.g., on YouTube, Instagram, Facebook, or TikTok)?”], intensity of social media use [“To what extent do you use social media (e.g., Instagram, TikTok, Facebook, or Twitter/X)?”], and frequency of online shopping [“To what extent do you purchase products or services online (e.g., via websites such as Amazon, Shein, or eBay)?”]. Items were rated on a 7-point scale ranging from 1 (*not at all*) to 7 (*very much*).

All measures were administered in Hebrew and were translated from the original English versions using a standard translation–back-translation procedure to ensure semantic and conceptual equivalence. Because the study focused on participants’ immediate post-exposure appraisals and evaluations, the primary measures were self-reported psychological and attitudinal responses. No behavioral indicators of advertising effectiveness were included in the present design.

#### 2.4.2. Narcissistic Admiration and Narcissistic Rivalry

Grandiose narcissism was assessed with the Narcissistic Admiration and Rivalry Questionnaire (NARQ) (Back et al., 2013). The NARQ includes 18 items (nine narcissistic admiration items and nine narcissistic rivalry items) rated on a 6-point Likert-type scale ranging from 1 (do not agree at all) to 6 (agree completely). Internal consistency in the present study was α = 0.82 for narcissistic admiration and α = 0.81 for narcissistic rivalry.

#### 2.4.3. Perceived Recognition (Post Manipulation)

Perceived recognition was assessed with an adapted version of the 6-item Perceived Recognition Scale (PRS; Besser et al., 2026), which operationalizes recognition as a self-relevant sense of being noticed, acknowledged, and valued. Items were adapted to the advertising context by replacing references to an interaction agent with references to the advertisement while preserving the original recognition-focused wording (e.g., “The ad made me feel personally seen or recognized,” “The ad seemed to value me as an individual”). Participants rated items on a 7-point scale (1 = strongly disagree, 7 = strongly agree). Items were averaged to create a composite score (α = 0.94). In prior work using the original PRS (Besser et al., 2026), CFA supported a single-factor structure and internal consistency was high (α = 0.93).

The development and validation of the original PRS are described in a separate validation study (Besser et al., 2026). In the present study, the PRS was used as an adapted state-level post-exposure measure in the advertising context. Although internal consistency was high, the present data do not by themselves provide a full within-sample validation of the adapted measure’s factorial and discriminant structure. Future work should therefore test the adapted version using CFA and related validity analyses within advertising samples.

#### 2.4.4. Perceived Freedom Threat and State Reactance (Post Manipulation)

Consistent with the combined mediator in Figure 1, resistance was assessed using two components–perceived freedom threat and state reactance–which were aggregated to represent a single resistance pathway. To compute the resistance index, scores for perceived freedom threat and state reactance were first standardized (z-scores) and then averaged, with higher values indicating stronger resistance. For robustness and interpretability, we also examined freedom threat and state reactance separately and observed the same pattern of conditional effects. This convergence supports treating the two components as closely related indicators of a broader autonomy-related resistance response for the primary observed-variable analyses. Accordingly, we report the composite index as the primary operationalization to preserve model parsimony and avoid redundancy across adjacent post-exposure measures. This approach is consistent with the theoretical sequence in which perceived freedom threat serves as a proximal trigger of state reactance, while treating both as a unified resistance pathway. Although perceived freedom threat is conceptually more proximal than state reactance, the use of a composite index reflects an analytic simplification of two closely linked post-exposure responses rather than a rejection of this sequence. Future research should examine this framework using serial mediation or latent-variable models that more explicitly preserve the threat-to-reactance progression. Accordingly, the separate analyses of perceived freedom threat and state reactance are not presented as primary models but should be interpreted cautiously and explored more fully in future work.

##### Perceived Freedom Threat

Perceived freedom threat was measured with the standard 4-item perceived threat to freedom measure (Dillard & Shen, 2005), capturing the extent to which the advertisement was perceived as pressuring or restricting personal choice. Responses were recorded on a 5-point Likert-type scale (1 = strongly disagree, 5 = strongly agree). Internal consistency in the present study was α = 0.80.

##### State Reactance

State reactance was measured using an adapted version of the Salzburger State Reactance Scale (SSR; Sittenthaler et al., 2015). The original SSR consists of 10 items designed to assess situational psychological reactance across three subdimensions: (1) experience of reactance (e.g., “I felt irritated”), (2) aggressive behavioral intentions (e.g., “I wanted to oppose the person who tried to influence me”), and (3) negative attitudes (e.g., “I had a negative opinion about the source”).

In the present study, all items were linguistically adapted to reflect reactions to a commercial advertisement rather than to a generic persuasive source (e.g., “I felt irritated by the advertisement,” “I wanted to do the opposite of what the ad suggested”). Participants rated their agreement with each statement on a 5-point Likert-type scale ranging from 1 (strongly disagree) to 5 (strongly agree). Higher scores indicated stronger state reactance toward the advertisement. An overall state reactance index was calculated by averaging all 10 items (α = 0.93 in the present study). The adapted version of the SSR has been used successfully in previous advertising-related studies and demonstrated high internal consistency (α ≈ 0.88–0.91; Bambauer-Sachse & Heinzle, 2018; Klein, 2020; Wehbe et al., 2017).

#### 2.4.5. Advertising Outcomes (Aad, Ab, PI)

Advertising outcomes were assessed using standard multi-item measures capturing attitudes toward the advertisement (Aad), attitudes toward the brand (Ab), and purchase intentions (PI).

##### Attitudes Toward the Advertisement (Aad)

Attitudes toward the advertisement (Aad) were assessed using three semantic differential items rated on a 7-point scale (Bad/Good; Unpleasant/Pleasant; Unfavorable/Favorable), as commonly used in advertising research (MacKenzie & Lutz, 1989). Internal consistency in the present study was α = 0.92.

##### Attitudes Toward the Brand (Ab)

Brand attitude (Ab) was assessed using the Spears and Singh (2004) measure. In the validated factor-analytic solution, five Ab items (Unappealing/Appealing; Bad/Good; Unpleasant/Pleasant; Unfavorable/Favorable; Unlikable/Likable) were retained as the brand attitude indicator set. Items were rated on a 7-point semantic differential scale. Internal consistency in the present study was α = 0.94.

##### Purchase Intentions (PI)

Purchase intentions (PI) were assessed using the Spears and Singh (2004) purchase intention measure, consisting of four items (Never/Whenever possible; Definitely do not intend to buy/Definitely intend to buy; Very low purchase interest/Very high purchase interest; Definitely will not buy/Definitely will buy). Items were rated on a 7-point semantic differential scale. Internal consistency in the present study was α = 0.97.

### 2.5. Design

A between-subjects experimental design was used. The manipulated independent variable was advertising strategy (recognition/validation vs. status-challenge). Narcissistic admiration and narcissistic rivalry served as focal predictors. Perceived recognition and the resistance index (perceived freedom threat and state reactance) served as mediators, and Aad, Ab, and PI served as outcomes. Consistent with the proposed model, we tested a conditional process (moderated mediation) framework in which advertising strategy was examined as a moderator of the associations between narcissistic traits and the mediators.

### 2.6. Ethical Considerations

All procedures complied with the Declaration of Helsinki and institutional ethical guidelines. The study was approved by the relevant institutional ethics committee (Approval Number: 0711; Approval Date: 31 December 2025). Participation was voluntary, and participants could withdraw at any time without penalty. The manipulation involved brief, commonplace advertising messages and posed no more than minimal risk. Participants were debriefed at the end of the study and provided with standard contact information for questions or concerns.

### 2.7. Statistical Analyses

Analyses began with descriptive statistics and Pearson correlations among study variables. The primary hypothesis tests used conditional process analyses to evaluate the proposed moderated mediation model, with advertising strategy entered as a dichotomous moderator. Condition was effect-coded (recognition/validation = −1; status-challenge = +1). Because PROCESS permits only a single focal predictor per model, narcissistic admiration and narcissistic rivalry were tested in two parallel models. In each model, one narcissism dimension served as the focal predictor, whereas the other dimension and its interaction with advertising strategy were included as covariates to account for shared variance and to hold constant any condition-contingent effect of the nonfocal dimension. Thus, focal effects were estimated in parallel rather than within a single omnibus model.

PROCESS was selected because the primary aim was to estimate observed-variable conditional indirect associations for each narcissism dimension while statistically accounting for the other dimension within a model structure closely aligned with the focal hypotheses. This approach provides a transparent test of the proposed indirect pathways at the observed-variable level. At the same time, we recognize that SEM would offer several advantages, including the simultaneous estimation of narcissistic admiration and rivalry pathways, explicit modeling of measurement error, formal comparison of indirect effects, and evaluation of overall model fit. Accordingly, the present analyses should be understood as observed-variable conditional process tests rather than as a full latent-variable modeling approach.

To evaluate potential multicollinearity, we examined variance inflation factors (VIFs) in the focal regression models. VIF values ranged from 1.02 to 1.10, indicating no evidence of problematic multicollinearity.

Perceived recognition and the composite resistance index (standardized perceived freedom threat and state reactance) were entered as mediators, and Aad, Ab, and PI were analyzed as outcomes. For each outcome, we estimated two parallel conditional process models (one with admiration as the focal predictor and one with rivalry as the focal predictor), yielding six focal models overall. Conditional indirect effects were estimated using bootstrapped confidence intervals with 10,000 resamples, and the index of moderated mediation was reported for each pathway along with conditional indirect effects within each advertising strategy condition. All exclusion rules, covariate decisions, and robustness checks were documented in the OSF repository (see Section 2.8). In the Results, models are reported in a fixed order: (a) regression terms and focal interactions, (b) bootstrapped indirect effects, and (c) the index of moderated mediation with conditional indirect effects by condition; model *R*^2^ and omnibus *F* statistics are provided in the corresponding tables.

### 2.8. Data Availability

To enhance transparency and reproducibility, the study materials are openly available on the Open Science Framework (OSF) at https://osf.io/6uekh (accessed on 4 January 2026). The repository includes (a) the anonymized dataset used for analysis, (b) the final video stimulus files for both strategy versions and narrator gender (four files), and (c) the full prompts/storyboard and production notes used to generate the stimuli.

## 3. Results

### 3.1. Background and Sociodemographic Variables

Table 1 summarizes the sociodemographic and background characteristics of the sample, presented for the full sample and stratified by gender and experimental condition. The reported variables include age, number of children, exposure to digital video advertisements, intensity of social media use, frequency of online shopping, educational attainment, employment status, marital status, household income, and religiosity. These characteristics are provided to describe the sample and to contextualize the experimental findings.

Between-condition comparisons were conducted using independent-samples t tests for continuous variables and *χ*^2^ tests for categorical variables. These analyses revealed no statistically significant differences between experimental conditions on any sociodemographic or background variable (all *p*s > 0.05). This pattern supports the internal validity of the experimental comparison by reducing the likelihood that observed condition effects are attributable to pre-existing group differences. Consequently, these variables were not included as covariates in the primary analyses.

To evaluate the potential confounding influence of narrator–gender matching, we conducted supplementary analyses examining participant gender in relation to the focal mediators and outcomes. These analyses indicated that participant gender was not associated with the mediators or outcomes, nor did it moderate the associations between narcissistic admiration or rivalry and the mediators or outcomes. In addition, gender did not moderate the indirect associations linking narcissistic admiration or rivalry to the outcomes through the mediators. Taken together, these results suggest that participant gender does not account for the observed pattern of findings; accordingly, it was not included in the primary analyses.

### 3.2. Univariate Analyses

The correlation coefficients and descriptive statistics can be found in Table 2. Narcissistic admiration exhibited small positive correlations with perceived recognition. In addition, narcissistic admiration showed small positive associations with attitudes toward the brand and purchase intentions in the recognition/validation condition; these associations did not emerge in the status-challenge condition.

Narcissistic rivalry demonstrated small-to-medium positive correlations with perceived recognition and the composite resistance index across both experimental conditions. Notably, narcissistic rivalry was moderately positively correlated with purchase intentions in the status-challenge condition, whereas this association was absent in the recognition/validation condition. As shown in Table 2, narcissistic admiration and narcissistic rivalry were only weakly correlated across conditions, which reduces concern regarding severe multicollinearity in the present models.

As shown in Table 3, participants in the recognition/validation condition did not differ from those in the status-challenge condition with respect to narcissistic admiration or narcissistic rivalry. This pattern indicates that random assignment was successful in producing comparable groups on these dispositional traits. As anticipated, participants in the recognition/validation condition reported significantly higher levels of perceived recognition than those in the status-challenge condition, supporting the effectiveness of the experimental manipulation. However, the two conditions did not differ on the composite resistance index, attitudes toward the advertisement, attitudes toward the brand, or purchase intentions.

Although the mean-level comparisons showed no significant differences between conditions for attitudes toward the advertisement, attitudes toward the brand, or purchase intentions (Table 3), the conditional process models estimate the effect of condition while simultaneously modeling perceived recognition and resistance. In this framework, a significant condition term reflects an adjusted direct effect that remains after accounting for the two mediators, rather than a contradiction of the unadjusted mean comparisons. Because recognition and resistance operate in opposing directions on outcomes, mean-level differences can be attenuated even when the indirect pathways are substantial.

### 3.3. Perceived Recognition and Composite Resistance Index

The results of the conditional process analysis showed that narcissistic admiration (*B* = 0.22, *CI_95%_*[0.14, 0.30], *SE* = 0.04, *t* = 5.53, *p* < 0.001) and narcissistic rivalry (*B* = 0.12, *CI_95%_*[0.05, 0.20], *SE* = 0.04, *t* = 3.12, *p* = 0.002) were positively associated with perceived recognition. Experimental condition was also a significant predictor such that participants in the recognition/validation condition reported higher perceived recognition than those in the status-challenge condition (*B* = 0.13, *CI_95%_*[0.05, 0.21], *SE* = 0.04, *t* = 3.33, *p* < 0.001). In contrast to H2, the association between narcissistic admiration and perceived recognition was not moderated by experimental condition (*B* = −0.04, *CI_95%_*[−0.12, 0.04], *SE* = 0.04, *t* = −0.96, *p* = 0.339). Similarly, the association that narcissistic rivalry had with perceived recognition was not moderated by experimental condition (*B* = 0.02, *CI_95%_*[−0.06, 0.10], *SE* = 0.04, *t* = 0.52, *p* = 0.605).

Narcissistic rivalry was positively associated with the composite resistance index (*B* = 0.20, *CI_95%_*[0.14, 0.27], *SE* = 0.04, *t* = 5.84, *p* < 0.001), whereas narcissistic admiration was not associated with the composite resistance index (*B* = −0.03, *CI_95%_*[−0.10, 0.04], *SE* = 0.04, *t* = −0.88, *p* = 0.379). Experimental condition was not associated with the composite resistance index (*B* = 0.02, *CI_95%_*[−0.05, 0.09], *SE* = 0.03, *t* = 0.70, *p* = 0.484) nor did it moderate the associations that narcissistic admiration (*B* = 0.01, *CI_95%_*[−0.06, 0.08], *SE* = 0.04, *t* = 0.22, *p* = 0.825) or narcissistic rivalry (*B* = 0.02, *CI_95%_*[−0.05, 0.09], *SE* = 0.04, *t* = 0.61, *p* = 0.545) had with it.

Table 4, Table 5 and Table 6 are organized using a parallel reporting structure to facilitate interpretation across models. Each table includes (a) the mediator model predicting perceived recognition, (b) the mediator model predicting the composite resistance index, and (c) the outcome model predicting attitude toward advertisement, attitude toward brand, or purchase intentions. Because the mediator models are the same across these analyses, they are reproduced in Table 4, Table 5 and Table 6 for completeness, including *R*^2^ values and omnibus *F* tests. The outcome models differ across tables only with respect to the specific dimension examined. Accordingly, the Results text focuses on hypothesis-relevant effects, including interaction terms where applicable, the bootstrapped indirect effects with 95% confidence intervals, and the index of moderated mediation.

### 3.4. Attitudes Toward the Advertisement

The results of the conditional process analysis predicting attitudes toward the advertisement are presented in Table 4. Neither narcissistic admiration (*B* = −0.02, *CI_95%_*[−0.08, 0.05], *SE* = 0.03, *t* = −0.50, *p* = 0.618) nor narcissistic rivalry (*B* = 0.02, *CI_95%_*[−0.04, 0.09], *SE* = 0.03, *t* = 0.72, *p* = 0.470) were associated with attitudes toward the advertisement. Experimental condition was associated with attitudes toward the advertisement (*B* = −0.07, *CI_95%_*[−0.13, −0.01], *SE* = 0.03, *t* = −2.28, *p* = 0.023) such that participants in the status-challenge condition reported more positive attitudes than those in the recognition/validation condition. Consistent with H1, perceived recognition was positively associated with attitudes toward the advertisement (*B* = 0.45, *CI_95%_*[0.39, 0.52], *SE* = 0.03, *t* = 13.82, *p* < 0.001). In contrast, the composite resistance index was negatively associated with these attitudes (*B* = −0.51, *CI_95%_*[−0.58, −0.43], *SE* = 0.04, *t* = −13.67, *p* < 0.001).

Narcissistic admiration demonstrated a positive indirect association with attitudes toward the advertisement through perceived recognition (*B* = 0.10, *CI_95%_*[0.06, 0.14], *SE* = 0.02, *z* = 5.02, *p* < 0.001), but not through the composite resistance index (*B* = 0.02, *CI_95%_*[−0.02, 0.05], *SE* = 0.02, *z* = 0.88, *p* = 0.378). Contrary to H3, experimental condition did not moderate the indirect association that narcissistic admiration had with attitudes toward the advertisement through perceived recognition (*B* = −0.03, *CI_95%_*[−0.11, 0.04], *SE* = 0.04) or the composite resistance index (*B* = −0.01, *CI_95%_*[−0.08, 0.06], *SE* = 0.04).

Narcissistic rivalry showed a more complex pattern. It demonstrated a positive indirect association with attitudes toward the advertisement via perceived recognition (*B* = 0.05, *CI_95%_*[0.02, 0.09], *SE* = 0.02, *z* = 2.90, *p* = 0.004), alongside a negative indirect association via the composite resistance index (*B* = −0.10, *CI_95%_*[−0.14, −0.07], *SE* = 0.02, *z* = −5.35, *p* < 0.001). However, inconsistent with H5, experimental condition did not moderate either indirect pathway for narcissistic rivalry through perceived recognition (*B* = 0.02, *CI_95%_*[−0.05, 0.09], *SE* = 0.04) or the composite resistance index (*B* = −0.02, *CI_95%_*[−0.09, 0.05], *SE* = 0.04).

**Table 4:** Results of the conditional process analysis for attitudes toward the advertisement (Aad).

	Outcome								
	M_1_: Perceived Recognition			M_2_: Composite Resistance Index			Y: Aad		
Predictor	*B*	*SE*	*p*	*B*	*SE*	*p*	*B*	*SE*	*p*
X_1_: Narcissistic Admiration (ADM)	0.22	0.04	<0.001	−0.03	0.04	0.379	−0.02	0.03	0.618
X_2_: Narcissistic Rivalry (RIV)	0.12	0.04	0.002	0.20	0.04	<0.001	0.02	0.03	0.470
M_1_: Perceived Recognition	–	–	–	–	–	–	0.45	0.03	<0.001
M_2_: Composite Resistance Index	–	–	–	–	–	–	−0.51	0.04	<0.001
W: Condition	0.13	0.04	<0.001	0.02	0.03	0.484	−0.07	0.03	0.023
X_1_ × W: ADM × Condition	−0.04	0.04	0.339	0.01	0.04	0.825	0.02	0.03	0.551
X_2_ × W: RIV × Condition	0.02	0.04	0.605	0.02	0.04	0.545	0.01	0.03	0.858
Constant	0.00	0.04	0.962	0.00	0.03	0.987	0.00	0.03	0.958
	*R*^2^ = 0.08			*R*^2^ = 0.06			*R*^2^ = 0.42		
	*F* = 10.58, *p* < 0.001			*F* = 7.01, *p* < 0.001			*F* = 60.48, *p* < 0.001		
Conditional Indirect Association of ADM with Aad through Perceived Recognition									
Condition		*Coeff.*		*Boot SE*		*Boot LCI*		*Boot UCI*	
Status-Challenge (−1)		0.12		0.03		0.06		0.18	
Recognition/Validation (+1)		0.08		0.03		0.03		0.14	
Conditional Indirect Association of ADM with Aad through Composite Resistance Index									
Condition		*Coeff.*		*Boot SE*		*Boot LCI*		*Boot UCI*	
Status-Challenge (−1)		0.02		0.03		−0.03		0.08	
Recognition/Validation (+1)		0.01		0.03		−0.04		0.06	
Conditional Indirect Association of RIV with Aad through Perceived Recognition									
Condition		*Coeff.*		*Boot SE*		*Boot LCI*		*Boot UCI*	
Status-Challenge (−1)		0.05		0.03		0.00		0.10	
Recognition/Validation (+1)		0.07		0.03		0.01		0.12	
Conditional Indirect Association of RIV with Aad through Composite Resistance Index									
Condition		*Coeff.*		*Boot SE*		*Boot LCI*		*Boot UCI*	
Status-Challenge (−1)		−0.09		0.03		−0.15		−0.04	
Recognition/Validation (+1)		−0.11		0.03		−0.17		−0.06	

### 3.5. Attitudes Toward the Brand

Table 5 summarizes the conditional process model predicting attitudes toward the brand. Neither narcissistic admiration (*B* = 0.00, *CI_95%_*[−0.07, 0.07], *SE* = 0.03, *t* = 0.04, *p* = 0.969) nor narcissistic rivalry (*B* = 0.00, *CI_95%_*[−0.07, 0.06], *SE* = 0.04, *t* = −0.12, *p* = 0.902) were associated with attitudes toward the brand. Experimental condition, however, was associated with attitudes toward the brand (*B* = −0.10, *CI_95%_*[−0.16, −0.03], *SE* = 0.03, *t* = −2.88, *p* = 0.004), with participants in the status-challenge condition reporting more favorable attitudes than those in the recognition/validation condition. Consistent with H1, perceived recognition was positively associated with attitudes toward the brand (*B* = 0.44, *CI_95%_*[0.38, 0.51], *SE* = 0.04, *t* = 12.63, *p* < 0.001). In contrast, the composite resistance index was negatively associated with these attitudes (*B* = −0.40, *CI_95%_*[−0.48, −0.32], *SE* = 0.04, *t* = −10.01, *p* < 0.001).

Turning to the indirect effects, narcissistic admiration was linked to more positive brand attitudes indirectly through perceived recognition (*B* = 0.09, *CI_95%_*[0.05, 0.13], *SE* = 0.02, *z* = 4.94, *p* < 0.001), but not through the composite resistance index (*B* = 0.01, *CI_95%_*[−0.02, 0.04], *SE* = 0.01, *z* = 0.88, *p* = 0.380). Contrary to H3, experimental condition did not moderate the indirect association that narcissistic admiration had with attitudes toward the brand through perceived recognition (*B* = −0.03, *CI_95%_*[−0.11, 0.04], *SE* = 0.04) or the composite resistance index (*B* = −0.01, *CI_95%_*[−0.07, 0.05], *SE* = 0.03).

Narcissistic rivalry showed opposing indirect associations with attitudes toward the brand. It demonstrated a positive indirect association with attitudes toward the brand via perceived recognition (*B* = 0.05, *CI_95%_*[0.02, 0.09], *SE* = 0.02, *z* = 2.89, *p* = 0.004), alongside a negative indirect association via the composite resistance index (*B* = −0.08, *CI_95%_*[−0.12, −0.05], *SE* = 0.02, *z* = −5.02, *p* < 0.001). However, inconsistent with H5, experimental condition did not moderate either indirect pathway for narcissistic rivalry through perceived recognition (*B* = 0.02, *CI_95%_*[−0.05, 0.09], *SE* = 0.03) or the composite resistance index (*B* = −0.02, *CI_95%_*[−0.07, 0.04], *SE* = 0.03).

**Table 5:** Results of the conditional process analysis for attitudes toward the brand (Ab).

	Outcome								
	M_1_: Perceived Recognition			M_2_: Composite Resistance Index			Y: Ab		
Predictor	*B*	*SE*	*p*	*B*	*SE*	*p*	*B*	*SE*	*p*
X_1_: Narcissistic Admiration (ADM)	0.22	0.04	<0.001	−0.03	0.04	0.379	0.00	0.03	0.969
X_2_: Narcissistic Rivalry (RIV)	0.12	0.04	0.002	0.20	0.04	<0.001	0.00	0.04	0.902
M_1_: Perceived Recognition	–	–	–	–	–	–	0.44	0.04	<0.001
M_2_: Composite Resistance Index	–	–	–	–	–	–	−0.40	0.04	<0.001
W: Condition	0.13	0.04	<0.001	0.02	0.03	0.484	−0.10	0.03	0.004
X_1_ × W: ADM × Condition	−0.04	0.04	0.339	0.01	0.04	0.825	0.01	0.03	0.842
X_2_ × W: RIV × Condition	0.02	0.04	0.605	0.02	0.04	0.545	0.03	0.03	0.394
Constant	0.00	0.04	0.962	0.00	0.03	0.987	0.00	0.03	0.932
	*R*^2^ = 0.08			*R*^2^ = 0.06			*R*^2^ = 0.33		
	*F* = 10.58, *p* < 0.001			*F* = 7.01, *p* < 0.001			*F* = 42.44, *p* < 0.001		
Conditional Indirect Association of ADM with Ab through Perceived Recognition									
Condition		*Coeff.*		*Boot SE*		*Boot LCI*		*Boot UCI*	
Status-Challenge (−1)		0.11		0.03		0.06		0.18	
Recognition/Validation (+1)		0.08		0.03		0.03		0.14	
Conditional Indirect Association of ADM with Ab through Composite Resistance Index									
Condition		*Coeff.*		*Boot SE*		*Boot LCI*		*Boot UCI*	
Status-Challenge (−1)		0.02		0.02		−0.02		0.06	
Recognition/Validation (+1)		0.01		0.02		−0.03		0.05	
Conditional Indirect Association of RIV with Ab through Perceived Recognition									
Condition		*Coeff.*		*Boot SE*		*Boot LCI*		*Boot UCI*	
Status-Challenge (−1)		0.05		0.02		0.00		0.10	
Recognition/Validation (+1)		0.06		0.03		0.01		0.11	
Conditional Indirect Association of RIV with Ab through Composite Resistance Index									
Condition		*Coeff.*		*Boot SE*		*Boot LCI*		*Boot UCI*	
Status-Challenge (−1)		−0.07		0.02		−0.12		−0.03	
Recognition/Validation (+1)		−0.09		0.02		−0.14		−0.05	

### 3.6. Purchase Intentions

Table 6 displays the conditional process model predicting purchase intentions. In this model, narcissistic rivalry emerged as a significant positive predictor of purchase intentions (*B* = 0.10, *CI_95%_*[0.04, 0.17], *SE* = 0.03, *t* = 3.03, *p* = 0.003), whereas narcissistic admiration was unrelated to purchase intentions (*B* = −0.02, *CI_95%_*[−0.08, 0.05], *SE* = 0.03, *t* = −0.46, *p* = 0.645). Experimental condition was also associated with purchase intentions (*B* = −0.08, *CI_95%_*[−0.14, −0.01], *SE* = 0.03, *t* = −2.37, *p* = 0.018), indicating that participants in the status-challenge condition reported stronger purchase intentions than those in the recognition/validation condition. Consistent with H1, perceived recognition was positively associated with purchase intentions (*B* = 0.60, *CI_95%_*[0.53, 0.66], *SE* = 0.03, *t* = 17.86, *p* < 0.001). In contrast, the composite resistance index was negatively associated with these intentions (*B* = −0.16, *CI_95%_*[−0.23, −0.09], *SE* = 0.04, *t* = −4.22, *p* < 0.001).

Narcissistic admiration demonstrated a positive indirect association with purchase intentions through perceived recognition (*B* = 0.13, *CI_95%_*[0.07, 0.18], *SE* = 0.03, *z* = 5.17, *p* < 0.001), but not through the composite resistance index (*B* = 0.01, *CI_95%_*[−0.01, 0.02], *SE* = 0.01, *z* = 0.84, *p* = 0.398). Contrary to H3, experimental condition did not moderate the indirect association that narcissistic admiration had with purchase intentions through perceived recognition (*B* = −0.05, *CI_95%_*[−0.15, 0.06], *SE* = 0.05) or the composite resistance index (*B* = 0.00, *CI_95%_*[−0.03, 0.02], *SE* = 0.01).

Narcissistic rivalry exhibited contrasting indirect associations. It demonstrated a positive indirect association with purchase intentions via perceived recognition (*B* = 0.07, *CI_95%_*[0.02, 0.12], *SE* = 0.02, *z* = 2.93, *p* = 0.003), alongside a negative indirect association via the composite resistance index (*B* = −0.03, *CI_95%_*[−0.05, −0.02], *SE* = 0.01, *z* = −3.40, *p* < 0.001). However, inconsistent with H5, experimental condition did not moderate either indirect pathway for narcissistic rivalry through perceived recognition (*B* = 0.02, *CI_95%_*[−0.07, 0.12], *SE* = 0.05) or the composite resistance index (*B* = −0.01, *CI_95%_*[−0.03, 0.02], *SE* = 0.01).

**Table 6:** Results of the conditional process analysis for purchase intentions (PI).

	Outcome								
	M_1_: Perceived Recognition			M_2_: Composite Resistance Index			Y: PI		
Predictor	*B*	*SE*	*p*	*B*	*SE*	*p*	*B*	*SE*	*p*
X_1_: Narcissistic Admiration (ADM)	0.22	0.04	<0.001	−0.03	0.04	0.379	−0.02	0.03	0.645
X_2_: Narcissistic Rivalry (RIV)	0.12	0.04	0.002	0.20	0.04	<0.001	0.10	0.03	0.003
M_1_: Perceived Recognition	–	–	–	–	–	–	0.60	0.03	<0.001
M_2_: Composite Resistance Index	–	–	–	–	–	–	−0.16	0.04	<0.001
W: Condition	0.13	0.04	<0.001	0.02	0.03	0.484	−0.08	0.03	0.018
X_1_ × W: ADM × Condition	−0.04	0.04	0.339	0.01	0.04	0.825	−0.03	0.03	0.425
X_2_ × W: RIV × Condition	0.02	0.04	0.605	0.02	0.04	00.545	0.06	0.03	0.078
Constant	0.00	0.04	0.962	0.00	0.03	0.987	0.00	0.03	0.921
	*R*^2^ = 0.08			*R*^2^ = 0.06			*R*^2^ = 0.40		
	*F* = 10.58, *p* < 0.001			*F* = 7.01, *p* < 0.001			*F* = 56.20, *p* < 0.001		
Conditional Indirect Association of ADM with PI through Perceived Recognition									
Condition		*Coeff.*		*Boot SE*		*Boot LCI*		*Boot UCI*	
Status-Challenge (−1)		0.15		0.04		0.08		0.23	
Recognition/Validation (+1)		0.11		0.04		0.04		0.18	
Conditional Indirect Association of ADM with PI through Composite Resistance Index									
Condition		*Coeff.*		*Boot SE*		*Boot LCI*		*Boot UCI*	
Status-Challenge (−1)		0.01		0.01		−0.01		0.03	
Recognition/Validation (+1)		0.00		0.01		−0.01		0.02	
Conditional Indirect Association of RIV with PI through Perceived Recognition									
Condition		*Coeff.*		*Boot SE*		*Boot LCI*		*Boot UCI*	
Status-Challenge (−1)		0.06		0.03		0.00		0.13	
Recognition/Validation (+1)		0.09		0.03		0.02		0.15	
Conditional Indirect Association of RIV with PI through Composite Resistance Index									
Condition		*Coeff.*		*Boot SE*		*Boot LCI*		*Boot UCI*	
Status-Challenge (−1)		−0.03		0.01		−0.05		−0.01	
Recognition/Validation (+1)		−0.04		0.01		−0.06		−0.02	

## 4. Discussion

The present study investigated how dimensions of grandiose narcissism shape responses to self-relevant video advertisements framed as either recognition/validation or status challenge. Grounded in the Narcissistic Admiration and Rivalry Concept (NARC; Back et al., 2013), we tested a dual-path process model in which two proximal mechanisms–perceived recognition and an autonomy-related resistance pathway (operationalized as a composite of perceived freedom threat and state reactance)–jointly account for consumer evaluations, including attitudes toward the advertisement, attitudes toward the brand, and purchase intentions. Across analyses, several consistent patterns emerged. More broadly, these findings advance identity-based persuasion research by demonstrating that affirmation-related and autonomy-protective processes can operate concurrently within the same advertising encounter and may partially offset one another at the aggregate level. At the same time, these findings should be interpreted with appropriate methodological caution. Because narcissistic admiration and rivalry were measured rather than manipulated, and because the mediators and outcomes were assessed within the same post-exposure session, the indirect associations are consistent with the proposed process model but do not establish causal mediation in a definitive temporal sense. In addition, several design and analytic choices place limits on the strength of the conclusions, including the use of expert-based rather than participant-level manipulation validation, the composite operationalization of resistance, and the reliance on observed-variable conditional process models rather than SEM.

At the mean level, recognition/validation framing increased perceived recognition relative to status challenge framing, supporting the effectiveness of the manipulation for the recognition construct. However, the two conditions did not differ in the resistance index or in consumer outcomes at the bivariate mean level, suggesting that framing alone did not uniformly shift downstream evaluations in this paradigm. The conditional process analyses clarified this pattern. Both narcissistic admiration and narcissistic rivalry were positively associated with perceived recognition, whereas only narcissistic rivalry was positively associated with the resistance index. In turn, perceived recognition was consistently and strongly associated with more favorable consumer responses, whereas the resistance index predicted less favorable responses. These proximal mechanisms produced the anticipated indirect effects: narcissistic admiration demonstrated a positive indirect pathway through perceived recognition, whereas narcissistic rivalry exhibited a competing dual-path pattern, with positive indirect effects via perceived recognition alongside negative indirect effects via resistance. Consistent with our decision to treat framing-based moderation as an empirical boundary condition rather than an assumption, we found no evidence that advertising strategy moderated the trait-to-mediator links or the indirect effects (see Section 4.5).

Taken together, these findings support a process-oriented account of self-relevant persuasion in which the same identity-targeted encounter can function as affirmation (when it elicits perceived recognition) and as an autonomy threat (when it elicits resistance), and in which narcissistic self-regulation helps explain who is more likely to engage each pathway. More specifically, the results suggest that self-relevant advertising is not persuasive simply because it is identity-linked; rather, its effectiveness depends on whether it is experienced as recognition or as an autonomy threat, and these processes are systematically related to distinct narcissistic self-regulatory tendencies.

At the same time, these conclusions should be interpreted within the specific context of the present experiment–a fictitious-brand, single-product, short-form video advertising paradigm conducted under tightly controlled exposure conditions. Whether this pattern generalizes across other product categories, real brands, media platforms, and advertising formats remains an open empirical question.

### 4.1. Self-Relevant Advertising as Meaning Based Persuasion

The effects of self-targeted advertising cannot be reduced to whether an advertisement is formally personalized or self-relevant. Rather, persuasion depends on the psychological meaning consumers assign to self-relevant cues. Classic and contemporary research shows that self-referencing increases elaboration and engagement, yet its downstream effects depend on how the self-connection is construed (Burnkrant & Unnava, 1995; Meyers-Levy & Peracchio, 1996; Escalas, 2007). In digital environments, this interpretive process is intensified because advertisements often signal knowledge of the consumer through personalization practices and behavioral targeting (Bang et al., 2019; Tucker, 2014). The same cue may be experienced as validating recognition or as an intrusive influence attempt that elicits skepticism and resistance, as emphasized by persuasion knowledge perspectives (Friestad & Wright, 1994) and research on online behavioral advertising (Baek & Morimoto, 2012; Edwards & Lee, 2002; Boerman et al., 2017, 2021).

The present findings align with this meaning-based view by showing that perceived recognition is a robust proximal predictor of favorable outcomes, above and beyond message condition and personality traits. In other words, recognition is not merely a design feature of the message. It is a recipient appraisal. This distinction sharpens both theory and practice because it clarifies why two consumers can be exposed to the same self-relevant message yet experience it in fundamentally different ways, with correspondingly divergent implications for persuasion and brand outcomes.

### 4.2. Recognition and Resistance as Separable Mechanisms

The results also support conceptualizing recognition and autonomy-related resistance as separable mechanisms rather than as opposite poles of a single response dimension. Psychological reactance theory posits that when individuals perceive a threat to their freedom, they experience a motivational state aimed at restoring autonomy, often expressed through anger, counterarguing, and negative evaluations of the message source (J. W. Brehm, 1966; Dillard & Shen, 2005). In contrast, Self-Determination Theory emphasizes that autonomy-supportive and affirming communication promotes receptivity and internalization (Deci & Ryan, 2000). Complementing this perspective, recent work underscores the importance of feeling “seen and valued” as a psychologically meaningful form of recognition that facilitates engagement and well-being (Weinstein et al., 2020). Viewed together, these frameworks suggest that self-relevant advertising can simultaneously invite validation and affiliation while also risking autonomy threat, particularly when the message is construed as pressuring, manipulative, or controlling (Dillard & Shen, 2005; Li & Shi, 2026; Miller et al., 2007; Quick & Stephenson, 2008).

Empirically, the present findings demonstrate that these two pathways have distinct antecedents and distinct consequences. Perceived recognition was consistently associated with more favorable attitudes and stronger purchase intentions, whereas the resistance index predicted less favorable consumer responses. Importantly, the absence of mean-level differences between conditions in resistance and consumer outcomes does not undermine the dual-mechanism account. Instead, it highlights why process modeling is essential in this domain. When recognition-related benefits and resistance-related costs co-occur within individuals, or when people differ in which pathway is activated, the mechanisms can partially offset each other in the aggregate, yielding small or nonsignificant mean-level condition differences even when the underlying pathways are active and predictive.

Although perceived freedom threat and state reactance are conceptually distinct, they are closely intertwined in persuasive contexts and frequently operate together. In the present study, these components are therefore treated as complementary indicators of autonomy-related resistance.

### 4.3. Narcissistic Self Regulation and the Recognition Pathway

The findings for narcissistic admiration are consistent with NARC theory and with a self-regulatory account of persuasion. Narcissistic admiration reflects an assertive, approach-oriented strategy characterized by charm, self-enhancement, and a desire for uniqueness and recognition (Back et al., 2013). In a self-relevant advertising context, these motives are likely to heighten attention to affirming cues and increase the likelihood that self-targeted content is appraised as recognition. The results aligned with this expectation: narcissistic admiration was positively associated with perceived recognition, and this link translated into more favorable consumer responses through a robust recognition-mediated pathway.

Equally important, narcissistic admiration did not predict the resistance index and did not show meaningful indirect effects through resistance. This pattern supports conceptualizing narcissistic admiration as primarily tied to recognition-oriented meaning making, rather than autonomy-restoration dynamics, in the present paradigm. It also clarifies why narcissistic admiration can be associated with more favorable persuasion outcomes in self-relevant contexts: not because narcissistic admiration directly increases liking for advertisements, but because it increases the likelihood that the encounter is construed as validating and affirming.

### 4.4. Rivalry as Competing Recognition and Resistance Processes

The pattern for narcissistic rivalry is particularly informative. Narcissistic rivalry reflects a defensive, antagonistic strategy characterized by entitlement, devaluation of others, hostility, and heightened sensitivity to ego threat and status competition (Back et al., 2013; Rogoza et al., 2019). Prior work suggests that narcissistic rivalry is linked to vigilance for status-relevant cues and heightened reactivity to perceived threats, which should increase the likelihood of autonomy-related resistance in response to persuasive attempts (Liu & Zheng, 2025). In the present study, narcissistic rivalry was positively associated with the resistance index, and this resistance pathway predicted less favorable consumer outcomes. This pattern supports the theoretically expected link between antagonistic narcissistic self regulation and autonomy-restoration dynamics in self-relevant persuasion contexts.

At the same time, narcissistic rivalry was also positively associated with perceived recognition. This is theoretically plausible and substantively important. Although narcissistic rivalry is defensive in orientation, it remains intensely self-referential and status-focused; in self-relevant contexts, status cues can be appraised as recognition cues even when the broader stance is suspicious or combative. As a result, the same exposure may yield a dual experience: the individual registers affirmation signals while also perceiving controlling intent or evaluative pressure. The indirect associations reflected exactly this profile, with narcissistic rivalry predicting more favorable outcomes indirectly via perceived recognition but less favorable outcomes indirectly via resistance. This competing dual-path pattern offers a process-level account for why narcissistic rivalry can sometimes appear “responsive” to self-relevant cues while simultaneously being linked to reactance and resistance.

In addition, narcissistic rivalry emerged as a positive predictor of purchase intentions in the purchase intentions model. One interpretation is that narcissistic rivalry may relate to purchase motivation through additional mechanisms not captured by perceived recognition or autonomy threat, such as competitiveness, dominance striving, or status-signaling consumption motives. This interpretation does not contradict the resistance pathway; rather, it suggests that for some consumers high in narcissistic rivalry, purchase intentions may reflect strategic self-presentation or status acquisition motives even while they experience autonomy threat and resistance. Future research is needed to test these additional pathways directly.

### 4.5. Advertising Strategy and the Absence of Moderated Mediation

A notable finding is that advertising strategy did not moderate either the trait-to-mediator paths or the indirect effects. Although recognition/validation framing increased perceived recognition at the mean level, the links from admiration and rivalry to perceived recognition and to the resistance index, as well as the downstream consequences of these mediators, were similar across both message framings. This absence of moderated mediation is theoretically informative because it suggests that narcissistic self-regulatory orientations may shape processing in a relatively stable manner across the two framing strategies tested here. It also implies that the persuasive “risk” associated with narcissistic rivalry is not confined to a particular style of self-relevance. Rather, narcissistic rivalry appears to be associated with a broader tendency toward autonomy-related resistance when persuasion is experienced as self-relevant. Notably, because the two advertisements were intentionally matched on overall autonomy-supportive tone, the manipulation primarily altered the strategic meaning of self-relevance rather than the degree of perceived interpersonal pressure. This design feature may help explain why the trait-to-appraisal links remained similar across conditions.

At the same time, the framing manipulation still mattered in that it shifted perceived recognition. This pattern supports treating framing as a lever that can raise average recognition appraisals without necessarily altering how personality traits map onto recognition and resistance. Practically, this suggests a clear boundary condition: message framing may move average appraisals in a favorable direction, yet personality-linked self-regulation can still shape whether resistance is activated when self-relevance is experienced as controlling.

The null moderation findings should be interpreted in light of statistical sensitivity. The study was powered a priori to detect small moderated-mediation effects, and the final sample exceeded this target. Accordingly, the absence of moderated mediation is unlikely to reflect insufficient statistical power. At the same time, very small interaction effects may still have gone undetected; thus, the findings are more consistent with weak or absent moderation in this paradigm than with strong condition-contingent trait effects.

### 4.6. Why Mean Level Effects Can Be Weak When Mechanisms Are Strong

The combination of null mean-level differences in consumer outcomes and strong process effects highlights an important methodological and conceptual point. Self-relevant advertising can activate countervailing mechanisms: perceived recognition supports persuasion, whereas autonomy-related resistance undermines it. When both mechanisms are present within a condition, or when individuals differ substantially in which pathway is most activated, total effects can cancel out at the aggregate level, producing small or nonsignificant mean-level differences even when the underlying processes are active. Conditional process modeling helps address this issue by partitioning these pathways and estimating indirect effects through each mechanism (Hayes, 2022). This underscores the importance of examining mediating mechanisms even when total effects are weak or nonsignificant. In the present study, this decomposition shows that perceived recognition and resistance are powerful predictors of outcomes, even when average condition differences in outcomes are modest.

This interpretation is also consistent with broader findings showing that reactance and freedom threat are reliable predictors of persuasion failure across contexts, even when mean effects of manipulations vary (Dillard & Shen, 2005; Rains, 2013; Li & Shi, 2026). The present contribution is to show that, in self-relevant advertising, recognition and resistance can be simultaneously activated and are systematically related to narcissistic self-regulation. In this sense, the present findings extend international research on persuasion and reactance by suggesting that variability in the effectiveness of self-relevant advertising may reflect not only message design, but also stable differences in how consumers regulate self-evaluation, recognition, and autonomy.

### 4.7. Practical Implications for Identity-Based and Personalized Marketing

Within the boundaries of the present single-product, short-form video paradigm, the findings have practical implications for designing self-targeted video advertising, especially in digital environments where personalization and algorithmic delivery are common. First, perceived recognition emerged as a strong and consistent lever for favorable consumer responses. This suggests that self-relevant messaging is more effective when it emphasizes acknowledgment and validation in ways that are experienced as autonomy-supportive rather than manipulative. Second, autonomy-related resistance is a consistent risk factor. When resistance is elicited, it reliably undermines attitudes and purchase intentions. Messages that are construed as controlling, pressuring, or evaluative are more likely to evoke perceived freedom threat and reactance (Miller et al., 2007; Quick & Stephenson, 2008).

These implications are consistent with research on persuasion knowledge, privacy, and personalization. When consumers infer that a message leverages their personal information to influence them, perceptions of manipulation and psychological reactance tend to increase (Boerman et al., 2017, 2021; Edwards & Lee, 2002; Friestad & Wright, 1994). In contrast, when personalization is accompanied by transparency, user control, and benevolent intent, trust is strengthened and reactance is reduced (Bleier & Eisenbeiss, 2015; Xu et al., 2025). Taken together, this literature suggests that the effectiveness of self-relevant advertising depends not only on whether personal cues are used, but on how they are framed and contextualized. This interpretation is also consistent with Self-Determination Theory, which emphasizes the persuasive value of autonomy-supportive communication (Deci & Ryan, 2000), and with reactance research showing that perceived threats to freedom undermine persuasion by eliciting resistance (Dillard & Shen, 2005; Miller et al., 2007). One applied implication is to design self-relevance as an autonomy-supportive invitation rather than as an evaluative prompt. Another is to pair identity-targeted cues with signals of choice and volition–for example, language that emphasizes optionality, personal agency, and alignment with the consumer’s own goals. Framing self-relevance in this manner may help preserve perceived recognition while minimizing autonomy-related resistance.

Although selling style was not manipulated directly, the results can be interpreted through a hard-sell versus soft-sell lens as a consumer-experience distinction rather than a strict message-format distinction. Soft-sell appeals are typically indirect and emotion- or image-based, whereas hard-sell appeals are more direct and explicit (Okazaki et al., 2010). In this framework, a self-relevant appeal may be experienced as “harder” when it is construed as controlling and autonomy-threatening, which elevates reactance, whereas it may be experienced as “softer” when it is construed as supportive recognition. Importantly, the current data do not show that status-challenge framing uniformly increased resistance. Instead, they indicate that resistance is strongly tied to individual differences, especially rivalry, and that once resistance is activated it consistently undermines persuasion outcomes.

Finally, the findings speak to personalization and segmentation. If advertisers aim to tailor self-relevant messages, the results suggest that the goal is not simply to increase self-relevance, but to increase perceived recognition while minimizing autonomy threat. This may be especially important for consumers who are more prone to antagonistic self-regulatory dynamics. At the same time, applied use of personality-based targeting raises ethical and transparency considerations. The most defensible implication is therefore not “target narcissism,” but “design self-relevance to reduce autonomy threat broadly,” which can improve persuasion effectiveness while respecting consumer agency.

### 4.8. Limitations

Several limitations should be acknowledged. First, the study relied on self-report measures of mediators and outcomes assessed shortly after a single exposure. This design limits inferences about long-term persuasion effects and real-world behavior, increases the risk of common method inflation, and constrains causal interpretation of the indirect pathways. In addition, the study did not include a marker variable, a latent common-method-factor model, or other formal statistical remedies for shared method variance.

Second, the manipulation was validated through independent expert ratings rather than participant-level manipulation checks. Although this reduced the risk of contaminating the focal mediators with conceptually overlapping items, it limits the ability to verify subjective construal directly at the participant level.

Third, resistance was operationalized as a composite index of perceived freedom threat and state reactance. Although this choice was analytically parsimonious and consistent with the proposed autonomy-related resistance pathway, it simplifies a theoretically sequential process and should therefore be interpreted cautiously.

Fourth, the study did not include behavioral indicators such as click-through, choice behavior, or actual purchase behavior, so the observed effects are limited to self-reported consumer evaluations.

Fifth, the manipulation was limited to a single product category and two specific short-form video advertisements. These stimuli were synthetically produced using a multi-platform AI pipeline to maximize realism and strict standardization (Section 2.2.3). While this approach strengthens experimental control and mirrors the increasing use of synthetic media in contemporary advertising, effects may differ when messages are produced and delivered by real brands with established reputations and source cues, which can shape perceived authenticity and trust. Message effects may also vary as a function of product category, product involvement, platform context, advertising format, and additional message features such as tone, directiveness, and transparency. Accordingly, the present findings should be generalized cautiously beyond the specific product and short-form video format used here.

Sixth, the study did not manipulate selling style directly. Any hard-sell versus soft-sell interpretation should therefore be treated as an interpretive lens that fits the observed pattern of mechanisms, rather than as a causal claim about selling style.

Seventh, narrator gender was matched to participant gender as a linguistic design constraint. Although this preserved naturalness in Hebrew, it also introduced a procedural feature that was not independently manipulated and therefore cannot be fully disentangled from participant gender in the present design.

Eighth, while random assignment produced comparable groups and supports internal validity, generalizability may depend on cultural context, advertising norms, and marketplace conditions. In addition, the sample was recruited through a combination of a local online panel and community channels and was not designed to be population-representative. This recruitment strategy may also introduce self-selection bias, as participation depended on voluntary enrollment and willingness to complete an online study. Accordingly, the findings should not be generalized beyond similar adult community samples without caution, and their applicability may vary across cultural, social, and marketplace contexts.

Finally, narcissistic admiration and rivalry were measured before exposure to the advertisement because they were conceptualized as antecedent traits. However, this ordering may have increased self-focus and thereby influenced subsequent processing of the message.

### 4.9. Future Research Directions

Future work can extend and refine these conclusions in several ways. First, research should incorporate behavioral outcomes (e.g., click-through, dwell time, sharing, actual purchase behavior), examine whether effects persist under repeated exposures, and test the proposed model in more naturalistic media contexts, including field experiments and digital environments characterized by distraction, message competition, and repeated ad exposure. Second, studies should test the resistance pathway by modeling perceived freedom threat and state reactance separately, to determine whether they have distinct antecedents and whether one component is more proximal to persuasion outcomes (Dillard & Shen, 2005; Steindl et al., 2015). Third, future work should broaden the set of potential moderators and boundary conditions that are central in the personalization and online behavioral advertising literature, including trust in the advertiser, persuasion knowledge, privacy concerns, and perceptions of transparency and user control (Bleier & Eisenbeiss, 2015; Boerman et al., 2017, 2021; Friestad & Wright, 1994; Tucker, 2014). Fourth, it will be important to examine whether rivalry’s positive direct association with purchase intentions reflects status-signaling motives or competitive arousal, which would require direct measurement of those constructs.

A further direction for research is to test message design features that more directly operationalize autonomy-supportive framing. For example, messages can be systematically varied in directiveness, use of imperative language, and explicitness of persuasive intent–features that have been shown to influence perceptions of control and levels of reactance (Miller et al., 2007; Quick & Stephenson, 2008; Rains, 2013). Manipulating these elements would allow for more precise tests of how linguistic and structural cues shape autonomy-related resistance within identity-based appeals.

In addition, future research should investigate how algorithmic personalization and AI-mediated recommendation systems influence perceived recognition and resistance, particularly under conditions of transparency versus opacity. Emerging evidence suggests that user control, explainability, and disclosure can reduce reactance and enhance trust in AI-enabled systems (Wang et al., 2023; Xu et al., 2025). Integrating these design features into experimental paradigms would clarify how technological framing interacts with psychological processes of validation and autonomy protection.

Taken together, the present findings therefore challenge one-dimensional accounts of self-relevant persuasion. They suggest that the effectiveness of identity-based appeals cannot be fully understood without simultaneously modeling recognition and resistance as parallel, and potentially competing, mechanisms. More broadly, future international research should test whether the present dual-path framework generalizes across cultural settings, media systems, and advertising norms, thereby clarifying the boundary conditions of self-relevant persuasion across markets.

## 5. Conclusions

This study advances consumer psychology by showing that self-relevant video advertising operates through two concurrent mechanisms, perceived recognition and autonomy-related resistance, which jointly shape attitudes toward the advertisement and brand, as well as purchase intentions. Narcissistic admiration primarily aligned with the recognition pathway, predicting more favorable consumer responses via heightened perceived recognition. Narcissistic rivalry showed a competing dual-path profile: it was linked both to perceived recognition and to autonomy-related resistance, yielding positive indirect effects via recognition alongside negative indirect effects via resistance.

These mechanisms were robust across recognition/validation and status-challenge framing, as moderated mediation was not supported. Taken together, the findings underscore that the effectiveness of self-targeted advertising depends less on self-relevance per se and more on whether consumers construe the encounter as recognition or autonomy threat. Practically, this highlights the value of designing self-relevant messages that strengthen perceived recognition while minimizing perceived control threats and reactance, particularly for consumers whose self-regulatory motives make resistance more likely.

## Figures and Tables

**Table 1:** Sociodemographic and background information.

		Men (*n* = 295)		Women (*n* = 303)		
		Experimental Condition				
	Total Sample (*N* = 598)	Recognition/Validation (*n* = 149)	Status-Challenge (*n* = 146)	Recognition/Validation (*n* = 155)	Status-Challenge (*n* = 148)	Recognition/Validation ≠ Status-Challenge
Age	42.83	43.56	46.01	42.21	39.62	*t* = 0.08
Number of children	1.90	2.13	2.02	1.90	1.56	*t* = 1.50
Exposure to digital video ads	4.94	4.77	4.63	4.98	5.36	*t* = −0.83
Intensity of social media use	4.82	4.50	4.56	5.02	5.18	*t* = −0.69
Frequency of online shopping	4.81	4.76	4.71	4.78	5.01	*t* = −0.65
Education						*χ*^2^ = 9.23
No high school degree	13.2%	18.8%	17.8%	11.0%	5.4%	
High school degree	23.9%	22.1%	24.0%	30.3%	18.9%	
Bachelor’s degree	41.8%	38.9%	39.0%	39.4%	50.0%	
Master’s degree	18.9%	17.4%	18.5%	15.5%	24.3%	
Ph.D. or equivalent	2.2%	2.7%	0.7%	3.9%	1.4%	
Employment						*χ*^2^ = 9.44
Full time	70.4%	77.2%	83.6%	63.2%	58.1%	
Part time	18.6%	12.1%	8.2%	24.5%	29.1%	
Unemployed	3.8%	4.7%	2.1%	4.5%	4.1%	
Going to school	2.0%	2.0%	2.1%	1.9%	2.0%	
Home maker	1.5%	1.3%	0.7%	1.9%	2.0%	
Retired	2.0%	2.0%	3.4%	0.6%	2.0%	
Marital Status						*χ*^2^ = 10.99
Single	15.9%	14.1%	12.3%	17.4%	19.6%	
Dating	6.7%	4.7%	6.2%	3.8%	12.2%	
Cohabiting	7.2%	7.4%	5.5%	8.4%	7.4%	
Married	62.0%	66.4%	66.4%	61.9%	53.4%	
Separated	0.5%	0.0%	1.4%	0.0%	0.7%	
Divorced	7.0%	6.0%	7.5%	8.4%	6.1%	
Widowed	0.7%	1.3%	0.7%	0.0%	0.7%	
Household income						*χ*^2^ = 1.89
Very high	14.4%	17.4%	23.3%	9.0%	8.1%	
Somewhat high	21.4%	25.5%	24.7%	16.8%	18.9%	
Moderate	26.9%	26.2%	30.1%	28.4%	23.0%	
Somewhat low	20.9%	14.8%	14.4%	25.8%	28.4%	
Very low	16.4%	16.1%	7.5%	20.0%	21.6%	
Religiosity						*χ*^2^ = 1.77
Secular	50.0%	47.7%	53.4%	48.4%	50.7%	
Traditional	22.6%	19.5%	24.7%	25.2%	20.9%	
Religious	13.5%	14.1%	8.2%	14.2%	17.6%	
Ultra-Orthodox	13.9%	18.8%	13.7%	12.3%	10.8%	

**Table 2:** Intercorrelations and descriptive statistics.

	1	2	3	4	5	6	7
1. Narcissistic Admiration	–	0.04	0.19 **	−0.02	0.10	0.10	0.08
2. Narcissistic Rivalry	0.06	–	0.15 *	0.25 ***	−0.02	0.00	0.21 ***
3. Perceived Recognition	0.27 ***	0.12 *	–	−0.11	0.48 ***	0.46 ***	0.63 ***
4. Composite Resistance Index	−0.03	0.21 ***	0.01	–	−0.51 ***	−0.43 ***	−0.21 ***
5. Attitudes Toward Advertisement	0.10	−0.02	0.47 ***	−0.41 ***	–	0.84 ***	0.61 ***
6. Attitudes Toward Brand	0.12 *	−0.05	0.46 ***	−0.31 ***	0.78 ***	–	0.65 ***
7. Purchase Intentions	0.17 **	0.09	0.60 ***	−0.08	0.57 ***	0.60 ***	–
*Mean_Recognition/Validation_*	3.61	2.12	2.96	0.02	4.48	4.34	2.90
*Standard Deviation_Recognition/Validation_*	0.83	0.74	1.46	0.89	1.36	1.33	1.40
*Skewness_Recognition/Validation_*	−0.15	0.62	0.52	0.70	0.12	0.18	0.35
*Kurtosis_Recognition/Validation_*	−0.17	−0.32	−0.31	−0.39	−0.39	−0.24	−0.78
*Mean_Status-Challenge_*	3.63	2.18	2.61	−0.02	4.55	4.47	2.92
*Standard Deviation_Status-Challenge_*	0.81	0.76	1.40	0.86	1.38	1.38	1.46
*Skewness_Status-Challenge_*	0.02	0.58	0.83	0.86	0.02	0.02	0.51
*Kurtosis_Status-Challenge_*	−0.13	−0.36	0.15	−0.07	−0.20	−0.42	−0.36

Note. The values below the diagonal are taken from participants in the recognition/validation condition, whereas the values above the diagonal are taken from participants in the status-challenge condition. * *p* < 0.05; ** *p* < 0.01; *** *p* < 0.001.

**Table 3:** Comparisons of the recognition/validation and status-challenge conditions.

	Recognition/Validation Condition (*n* = 304)		Status-Challenge Condition (*n* = 294)		
	*M*	*SD*	*M*	*SD*	*t*
Narcissistic Admiration	3.61	0.83	3.63	0.81	−0.31
Narcissistic Rivalry	2.12	0.74	2.18	0.76	−0.97
Perceived Recognition	2.96	1.46	2.61	1.40	3.04 ***
Composite Resistance Index	0.02	0.89	−0.02	0.86	0.47
Attitudes Toward Advertisement	4.48	1.36	4.55	1.38	−0.62
Attitudes Toward Brand	4.34	1.33	4.47	1.38	−1.20
Purchase Intentions	2.90	1.40	2.92	1.46	−0.22

*** *p* < 0.001.

## Data Availability

See Section 2.8 for details regarding the OSF repository (anonymized dataset, video files, and full text prompts).
